# Cognitive correlates of abnormal myelination in psychosis

**DOI:** 10.1038/s41598-019-41679-z

**Published:** 2019-03-26

**Authors:** Lucy D. Vanes, Elias Mouchlianitis, Erica Barry, Krisna Patel, Katie Wong, Sukhwinder S. Shergill

**Affiliations:** 1 Institute of Psychiatry, Psychology and Neuroscience, de Crespigny Park, London, SE5 8AF United Kingdom; 2 Wellcome Centre for Human Neuroimaging, University College London, London, WC1 3BG United Kingdom

## Abstract

Psychotic illness has consistently been associated with deficits in cognitive function and reduced white matter integrity in the brain. However, the link between white matter disruptions and deficits in cognitive domains remains poorly understood. We assessed cognitive performance and white matter myelin water fraction (MWF) using multicomponent driven equilibrium single pulse observation of T1 and T2 (mcDESPOT) in recent-onset psychosis patients and age-matched healthy controls (HC). Psychosis patients showed deficits in working memory, phonological and semantic fluency, general intelligence quotient and reduced MWF in the left temporal white matter compared to HC. MWF in the left inferior fronto-occipital fasciculus and inferior longitudinal fasciculus was positively associated with intelligence quotient and verbal fluency in patients, and fully mediated group differences in performance in both phonological and semantic verbal fluency. There was no association between working memory and MWF in the left temporal white matter. Negative symptoms demonstrated a negative association with MWF within the left inferior and superior longitudinal fasciculi. These findings indicate that psychosis-related deficits in distinct cognitive domains, such as verbal fluency and working memory, are not underpinned by a single common dysfunction in white matter connectivity.

## Introduction

Cognitive deficits are a core characteristic of psychotic illness and a major contributor to the disability experienced by affected individuals^1–3^. Impaired performance spans a wide range of cognitive domains and is typically resistant to treatment with antipsychotic medication. Therefore, identifying the underlying neurobiology of cognitive deficits in psychosis remains an important objective which could lead to the development of improved treatment strategies for these dysfunctions.

White matter abnormalities in psychosis and schizophrenia have been consistently reported in the neuroimaging literature^4–6^. These abnormalities include reduced white matter volume as well as fractional anisotropy (FA) derived from diffusion tensor imaging (DTI) across the brain in patients compared to healthy controls, with particularly pronounced reductions in left fronto-temporal regions. These findings are typically attributed to abnormal myelination of connecting fibres in the brain, hampering efficient communication between distinct brain regions and thus leading to a range of psychotic and cognitive symptoms^4^.

Indeed white matter abnormalities have been shown to be associated with specific cognitive deficits in schizophrenia^7^. For example, executive function deficits were found to correlate with reduced FA in the anterior cingulate, corpus callosum^8^ and fornix^9^. Psychomotor speed was selectively related to FA in the corpus callosum, internal capsule, superior corona radiata and superior longitudinal fasciculus in patients with schizophrenia, but not in healthy control subjects^10^. Reduced FA in several other regions has been shown to correlate with impaired attention^8,11^, memory^8,12^ and general intelligence^12,13^. As yet there is a lack of consistency in terms of regions showing relevance for cognitive functioning in schizophrenia, although it is clear that white matter integrity plays an important role in cognition both in health and psychosis in general^7^. Although cognitive deficits have been suggested to be generalised across performance domains^14^, it is unlikely that a single neurobiological dysfunction underlies all domain-specific cognitive deficits. As such, separable impairments in different cognitive domains may be linked with distinct aspects of psychotic symptoms and also benefit from different treatment options.

Recent research on white matter in psychosis has most commonly utilised DTI methods and attributed findings of abnormal FA to disruptions in myelination^4^. However, it has been shown that FA is not selectively sensitive to changes in myelination and may therefore reflect a number of further factors, such as axonal membrane integrity, fibre density, axon diameter or number^15–17^. Findings from diffusion studies can therefore be usefully complimented by research utilising alternative myelin imaging methods such as multicomponent relaxometry techniques, which are more sensitive to changes in myelination itself^18,19^. Multicomponent driven equilibrium single pulse observation of T1 and T2 (mcDESPOT) is one such technique, from which whole brain voxelwise myelin water fraction (MWF) can be derived^20^. MWF indexes the fraction of water protons within myelin sheaths relative to protons in intra-/extracellular space and cerebrospinal fluid. As such, MWF provides a quantitative measure of myelination which is less affected by many of the factors influencing FA. In a recent study, we compared whole-brain myelin water fraction (MWF) derived using multicomponent driven equilibrium single pulse observation of T1 and T2 (mcDESPOT) in patients with chronic schizophrenia and healthy control subjects^21^. Patients showed reduced MWF in bilateral subcortical and temporal regions including the inferior fronto-occipital fasciculus. Moreover, MWF in the corpus callosum was shown to mediate behavioural differences between patients and controls in a cognitive inhibition task. McDESPOT was therefore shown to be a useful technique to identify changes in myelin content which are related to cognitive outcomes in chronic schizophrenia. However, it is not possible to separate the effects of age, chronicity of illness and the impact of long term medication in this latter study.

The aim of the current study is to compare white matter MWF in a sample of recent-onset psychosis patients with that in healthy control subjects and to assess how specific changes in myelin content relate to cognitive deficits in this early phase of the illness. Due to critical effects of ageing on brain myelination^22,23^ we conduct comparative analyses within age-matched subsamples of patients (N = 35) and controls (N = 35). We then test for associations with performance on a range of cognitive tasks (general intelligence, working memory, and verbal fluency) in patients with psychosis (N = 82) in order to assess whether the regions showing abnormal myelin content can generally account for variability in cognitive function in patients. Finally, mediation analyses are conducted in the age-matched subsamples in order to assess whether abnormal myelination can fully account for group differences observed in cognitive performance. We tested the hypothesis that there would be reductions in MWF in psychosis patients consistent with the current DTI literature on early psychosis, prominently in left fronto-temporal regions, which in turn would be associated with deficits in cognitive performance.

## Methods and Materials

### Participants

87 patients with recent onset psychosis and 35 healthy controls (HC) were included in the current study. Patients had experienced a first psychotic episode within the last 5 years (mean illness duration = 1.6 years). Exclusion criteria for all participants were a history of neurological illness, current major physical illness, and diagnosed drug dependency over the past six months. HC did not have a history of psychiatric illness or a first-degree relative currently or previously suffering from a psychotic illness. Neuroimaging data from a subset of the HC participants have previously been presented elsewhere^21^. All subjects provided written informed consent to take part the study and were compensated for their time and travel. Ethical approval was obtained by the Camberwell and St. Giles NHS National Research Ethics Committee and experiments were compliant with the Declaration of Helsinki.

Due to differences in age and sample size between psychosis patients and HC, a subsample of patients was selected using the MatchIt package in R (using age and sex as matching variables). This resulted in size and age-matched subsamples of 35 patients and 35 controls. Sample characteristics for the full samples, as well as the matched subsamples can be found in Table 1.

**Table 1:** Sample characteristics of full psychosis patient sample, age-matched patient subsample and healthy control sample, and statistical comparisons between age-matched groups.

	Patients (N = 82)		Patients (N = 35)		Controls (N = 35)			
	M	SD	M	SD	M	SD	Statistics	
							χ^2^(1)	P
Female (%)	37%		38%		34%		0.06	0.803
							t(68)	P
Age	26.8	6.2	32.5	4.9	35.1	9.8	1.39	0.170
NS-SEC			2.8	1.7	2.8	1.6	0.10	0.919
Onset age (years)	25.3	6.3	30.9	5.3				
Illness duration (years)	1.6	1.1	1.6	1.1				
CPZ equivalents	248.9	148.8	253.2	138.2				
**PANSS score**								
Positive symptoms	12.7	5.2	13.3	6.1				
Negative symptoms	12.8	5.5	11.9	4.9				
General symptoms	29.23	7.8	29.4	8.4				
Total score	54.8	15.5	54.6	16.4				

Abbreviations: NS-SEC, National Statistics Socio-economic Classification; CPZ, Chlorpromazine; PANSS, Positive and Negative Symptom Scale.

### Assessments

Participants underwent behavioural clinical assessments on the same day as undergoing MRI scanning. Intelligence quotient (IQ) was measured using the two-subtest Wechsler Abbreviated Scale of Intelligence (WASI; Wechsler, 1999^24^), consisting of the matrix reasoning and vocabulary subtests. Working memory was assessed using the letter-number-sequencing task, in which participants were required to recall a sequence of letters and numbers and rearrange them into ascending and alphabetical order. Verbal fluency was measured as the mean number of words participants were able to produce within one minute beginning with a particular letter (phonological verbal fluency; letters A, F, and S) or belonging to a particular category (semantic verbal fluency; categories animals and fruit). Symptoms were assessed in patients using the Positive and Negative Syndrome Scale (PANSS), administered by a trained researcher.

### MRI acquisition and processing

Scans were acquired on a 3T GE Excite II MR scanner (GE Healthcare, USA) with an 8-channel head coil. The mcDESPOT protocol consisted of a spoiled gradient recalled echo (SPGR) sequence across nine flip angles α (TR = 8.0 ms; TE = 3.6 ms; α = [2, 3, 4, 5, 6, 7, 9, 13, 18]°; matrix = 128 × 128), and a balanced steady-state free procession (bSSFP) sequence across eight flip angles, acquired at radiofrequency phase cycling patterns of 0° and 180° to correct for off-resonance effects (TR = 3.8 ms; TE = 1.9 ms; α = [12, 16, 21, 27, 33, 40, 51, 68]; matrix = 128 × 128). In addition an inversion recovery SPGR was acquired for estimating the flip angle error caused by transmitting B1 field inhomogeneity (TR = 8.0 ms; TE = 3.6 ms; α = 5°; matrix = 220 × 110). The field of view was 220 × 220 cm and the voxel size was 1.7 mm isotropic. The full mcDESPOT sequence lasted approximately 13 minutes.

Each subject’s scans were linearly coregistered, and non-brain parenchyma signal removed using BET (FMRIB Software Library, www.fmrib.ox.ac.uk/fsl). McDESPOT maps were processed using previously described open-source software^25^. Briefly, T1 & B1 maps were first calculated from the data^26^, followed by T2 & off-resonance maps^27^. Finally, Myelin Water Fraction (MWF) maps were derived using a three component model and Gaussian Region Contraction^28^. MWF maps were non-linearly registered to the MNI152 2 mm isotropic standard brain, and smoothed using a Gaussian kernel at 5 mm FWHM. All raw images were visually inspected for motion artefacts and datasets with artefacts excluded from all further analyses, resulting in the exclusion of 5 patients.

### Statistical analysis

In subsequent analyses, age matched subsamples (total N = 70) were used for all analyses drawing a direct comparison between patients and controls (including mediation analysis). The larger size of the psychosis patient sample (N = 82) was exploited for within-patient analyses of associations between cognitive performance and MWF.

Patients and controls were compared in terms of WASI IQ, letter-number-sequencing, phonological verbal fluency, and semantic verbal fluency using independent samples t-tests.

All analyses on MWF data were performed using non-parametric permutation tests with FSL’s randomise (with 10000 permutations). Significance values were corrected for multiple comparisons using threshold-free cluster enhancement (TFCE) and clusters were defined with an extent threshold of 10 voxels.

In order to first identify regions of abnormal white matter MWF in patients, we assessed the effect of group (patients vs. controls) on MWF in the age-matched subsamples. Age and gender were included as additional covariates of non-interest. A binary mask encompassing all tracts in the JHU white matter tractography atlas^29^ thresholded at 10% was used so as to restrict the analysis to white matter tracts only. The resulting significant cluster showing reduced MWF in patients compared to healthy controls was used as a region of interest (ROI) for all following analyses in order to investigate the association of myelination in this region with cognition and symptoms of patients.

Within the ROI, voxelwise correlation analyses were conducted using FSL randomise across the full patient sample between MWF and cognitive measures, controlling for age and gender. We did this in order to assess specifically whether abnormal myelin content was related to cognition in patients with psychosis. Separate analyses were run for WASI IQ, letter-number-sequencing, phonological verbal fluency, and semantic verbal fluency. We also tested for correlations within the ROI between MWF and PANSS negative, positive, and total scores, respectively, controlling for age and gender, where data was available (N = 81). Finally, we tested for correlations between MWF and CPZ equivalent dosages (N = 77), controlling for age and gender.

While the analyses described so far can establish whether groups differ in terms of cognition and myelin content, and whether there is an association between abnormal myelin and cognitive deficits, it does not directly address the issue of whether myelin lies on the causal pathway between diagnostic group and cognition. We therefore conducted mediation analyses in order to ascertain whether abnormal MWF (in the regions identified as significantly associated with cognition in psychosis patients) could statistically account for group differences on that particular cognitive measure. Mediation relies on an initial association between the independent (group) and dependent (cognition) variable, as well as between the mediator (MWF) and dependent variable. We therefore only performed mediation analyses for cognitive variables which showed a significant association with MWF in the previous analysis.

Significance of the mediating effect is tested by means of the indirect effect of group on cognitive performance (defined as the product between the effect of group on MWF and the effect of MWF on cognitive performance, controlling for group). A bootstrap approach with 5000 bootstrap samples was used in order to test for significance of the indirect effect using the PROCESS macro in SPSS^30^. Complete mediation is said to have occurred when a significant effect of group on cognition is rendered non-significant after including MWF as an additional independent variable.

## Results

### Group differences in cognitive measures

Cognitive scores of 35 HC and 35 age-matched psychosis patients were compared using independent samples t-tests. Patients showed lower WASI IQ scores (*M* = 98.2, *SD* = 16.5) compared to HC (*M* = 117.6, *SD* = 11.5), *t*(68) = 5.71, *p* < 0.001. Patients also scored lower on the letter-number-sequencing task (*M* = 10.0, *SD* = 2.1) compared to HC (*M* = 11.8, *SD* = 3.1), *t*(68) = 2.90, *p* = 0.005. Phonological verbal fluency was lower in patients (*M* = 11.6, *SD* = 4.5) compared to HC (*M* = 14.4, *SD* = 3.6), *t*(68) = 2.61, *p* = 0.011; as was semantic verbal fluency in patients (*M* = 15.9, *SD* = 4.3) compared to HC (*M* = 19.5, *SD* = 4.2), *t*(68) = 3.54, *p* = < 0.001.

### Group differences in MWF

**Figure 1:**
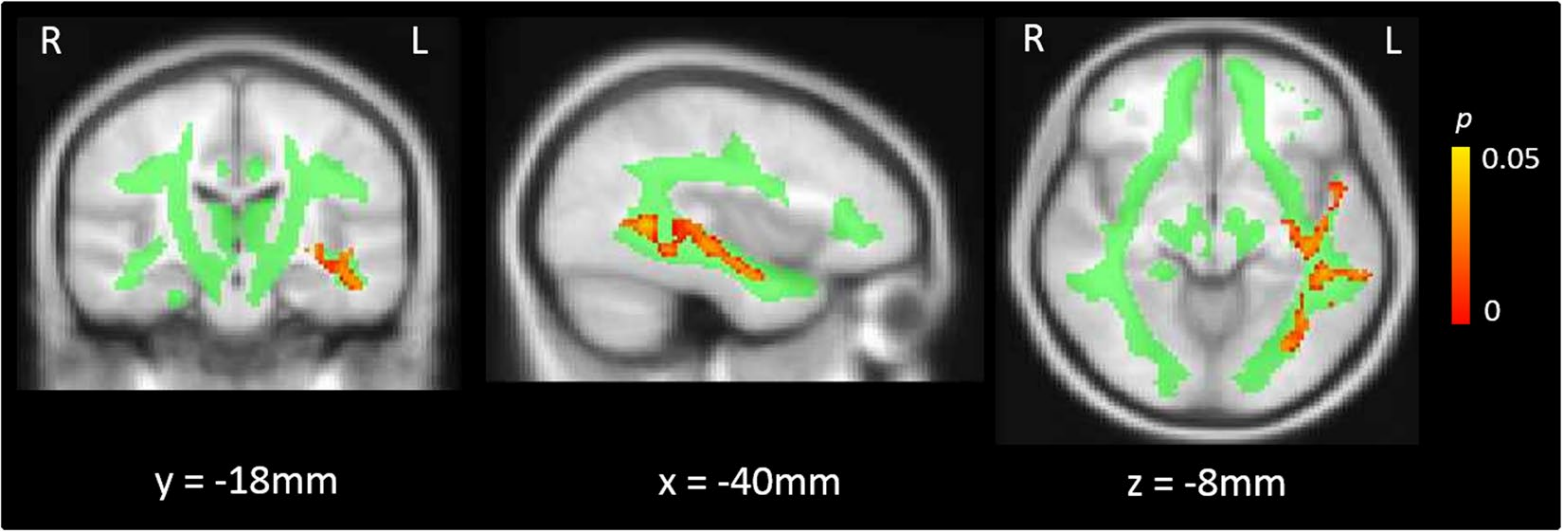
Regions of significantly reduced myelin water fraction in recent-onset psychosis patients compared to healthy controls (red), overlaid on the JHU white matter mask (green) used for analysis.

A non-parametric test of the effect of group on MWF, controlling for age and gender, yielded a significant cluster in the left temporal deep white matter (MNI: *X* = −46, *Y* = −50, *Z* = 2, 1200 voxels, *p* = 0.010) primarily encompassing the inferior longitudinal fasciculus, inferior fronto-occipital fasciculus and aspects of the superior longitudinal fasciculus. Within this region, psychosis patients had significantly reduced MWF values compared to age-matched HC (Fig. 1).

### Associations between MWF and cognitive measures in psychosis patients

**Figure 2:**
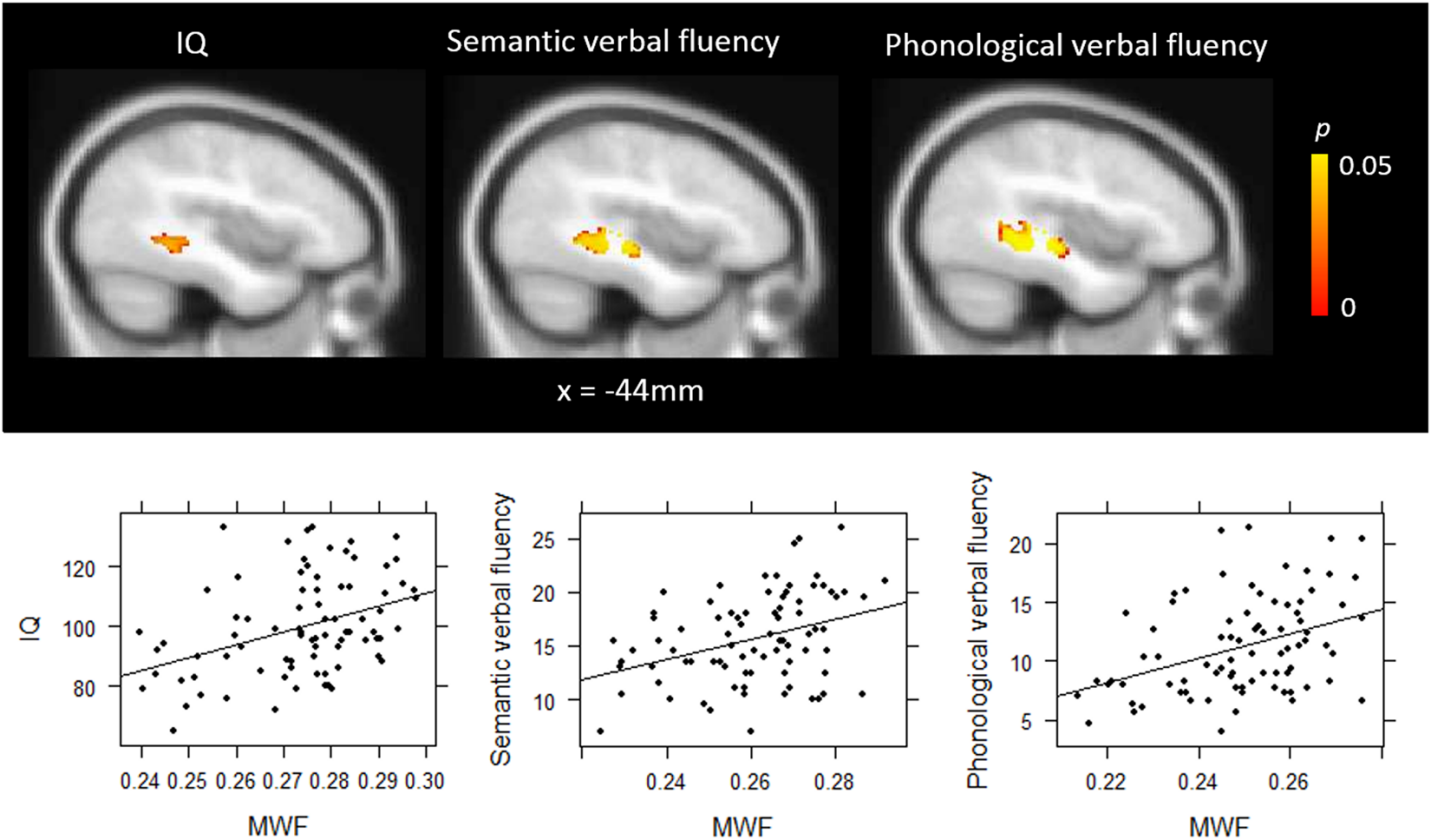
Significant associations between myelin water fraction and intelligence quotient (IQ), semantic verbal fluency and phonological verbal fluency.

We next tested for correlations between cognitive measures and MWF within the ROI showing reduced MWF in patients. Correlations were assessed in the full patient sample controlling for age and gender using randomise. There were significant positive correlations between MWF and WASI IQ (MNI: *X* = −42, *Y* = −42, *Z* = −8, 136 voxels, *p* = 0.013), phonological verbal fluency (MNI: *X* = −40, *Y* = −40, *Z* = −10, 462 voxels, *p* < 0.001) and semantic verbal fluency (MNI: *X* = −42, *Y* = −26, *Z* = −6, 298 voxels, *p* = 0.003) in overlapping regions within the inferior fronto-occipital fasciculus and inferior longitudinal fasciculus (Fig. 2). There was no significant association between performance on the letter-number-sequencing task and MWF.

To examine whether the correlation between WASI IQ and MWF was driven by one of the subtest scores, we extracted mean MWF values from the cluster showing a significant relationship with WASI IQ total score and conducted correlation analyses with the vocabulary subtest and the matrix reasoning subtest t-scores, respectively. MWF was significantly correlated with both vocabulary scores (*R* = 0.25, *p* = 0.027) and matrix reasoning scores (*R* = 0.47, *p* < 0.001).

### Associations between MWF and clinical measures in psychosis patients

There was a significant negative association between MWF and negative symptom severity in two small clusters in the anterior portion of the inferior longitudinal fasciculus (*X* = −48, *Y* = −16, *Z* = −22, 21 voxels, *p* = 0.036) and in the superior longitudinal fasciculus (*X* = −40, *Y* = −52, *Z* = 4, 15 voxels, *p* = 0.027) indicating that decreased MWF was associated with increased negative symptom severity in these regions. There were no significant associations between MWF and PANSS positive or total symptom scores or with CPZ equivalent medication dosage. Given the relevance of the inferior longitudinal fasciculus in verbal and visual hallucinations, we conducted a post-hoc correlative analysis specifically for the hallucinations subscore of the PANSS. There was no significant correlation between MWF and severity of hallucinations in this region.

### Mediation analyses

We extracted the mean MWF values from each cluster showing a significant correlation between MWF and WASI IQ, phonological verbal fluency, and semantic verbal fluency for all subjects in the age-matched subsamples (referred to as MWF_IQ_, MWF_Phon_, and MWF_Sem_ in the following). Mediation analyses were then conducted in order to ascertain whether reduced MWF in these regions significantly accounted for the group differences in these cognitive variables.

For WASI IQ, there was a significant effect of group on IQ, *β* = −0.013, *p* < 0.001. When MWF_IQ_ was included in the model, there was a significant effect of MWF_IQ_ on IQ, *β* = 263.32, *p* < 0.035. However, the effect of group remained significant and in fact increased to *β* = −15.99, *p* < 0.001. This indicates that MWF in the inferior fronto-occipital fasciculus is not a mediator of group differences in IQ.

There was a significant effect of group on phonological verbal fluency, *β* = −0.02, *p* < 0.001. After inclusion of MWF_Phon_, there was a significant effect of MWF_Phon_ on phonological fluency, *β* = 122.67, *p* < 0.001, and the effect of group was rendered non-significant (*β* = −1.04, *p* = 0.360). Non-parametric bootstrapping^30^ of the indirect effect (defined as the product between the effect of group on MWF_Phon_ and the effect of MWF_Phon_ on phonological fluency, controlling for group) with 5000 bootstrap samples revealed a significant indirect effect (95% confidence interval [−3.27, −0.60]), indicating that group differences in phonological fluency are fully mediated by MWF differences in the inferior fronto-occipital fasciculus.

**Figure 3:**
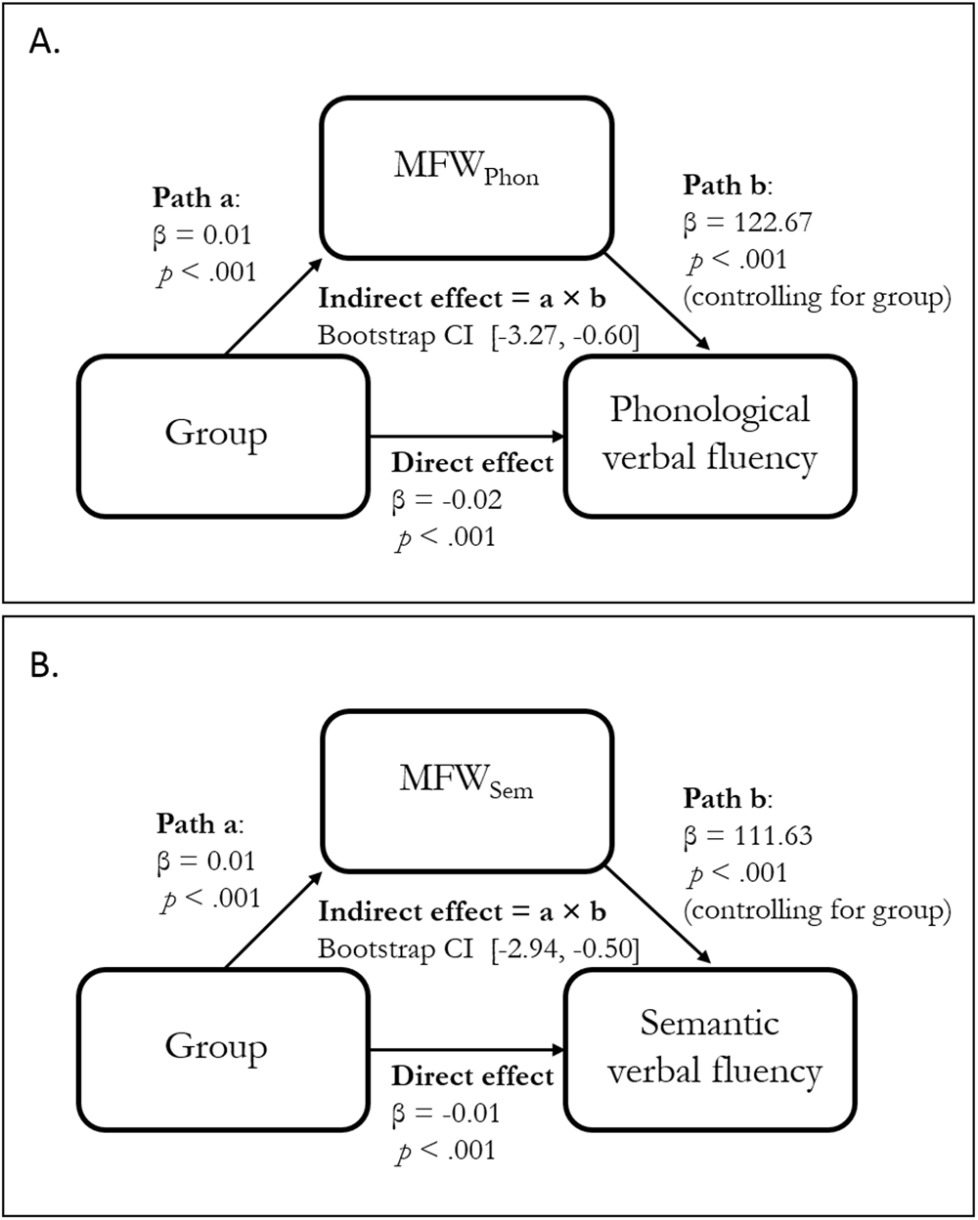
Schematic mediation diagram of the association between group, myelin water fraction (MWF), and phonological verbal fluency (**A**) and semantic verbal fluency (**B**).

Lastly, there was a significant effect of group on semantic fluency, *β* = −0.01, *p* < 0.001. After including MWF_Sem_, there was a significant effect of MWF_Sem_ on semantic fluency, *β* = 111.63, *p* = 0.001, but the effect of group was rendered non-significant, *β* = −2.04, *p* = 0.058. Bootstrap sampling revealed a significant indirect effect (95% confidence interval [−2.94, −0.50]), indicating that group differences in MWF_Sem_ fully mediate group differences in semantic fluency. A schematic diagram of the relevant pathways involved in the significant mediations is represented in Fig. 3.

## Discussion

Despite the fact that diagnostic criteria for psychosis and schizophrenia do not explicitly incorporate cognitive dysfunction, cognitive deficits are a demonstrable and debilitating feature of psychotic illness^31^. The underlying pathology of cognitive impairments is yet to be clarified, but structural connectivity of white matter tracts in the brain is thought to play an important role. In this study, we aimed to assess whether abnormal myelination of white matter tracts in psychosis is associated with domain-specific deficits in cognitive functioning and can fully account for differences seen compared to healthy control subjects.

We found significant deficits in recent-onset psychosis patients compared to age-matched healthy controls (HC) on general IQ, working memory, phonological verbal fluency and semantic verbal fluency. We also observed significantly reduced myelin water fraction (MWF) in the left temporal deep white matter in patients compared to age-matched HC. Crucially, within this region, reduced MWF in psychosis patients fully mediated group differences in both phonological and verbal fluency. Although there was an association between MWF and IQ, it did not fully account for the deficits in IQ observed in the patient sample. In contrast, working memory performance was not associated with myelin content in the left temporal deep white matter. Given our primary interest in the cognitive correlates of myelin in psychosis patients in regions showing myelin abnormalities, this study does not include whole-brain analyses of cognitive correlates of myelin in the HC group. Evidence of associations between cognitive functioning and measures of myelination in HC is reviewed in MacKay and Laule (2016)^32^.

Our findings of reduced MWF are in line with the current diffusion imaging literature indicating that there are consistent FA reductions in psychosis and schizophrenia in the left temporal lobe^5,6^. Given the involvement of left hemispheric temporal regions in language processing and production^33,34^, pathological changes in these areas are thought to be involved in the development of auditory verbal hallucinations^35,36^. Schizophrenia has been associated with increased temporal activation both at rest and during hallucinatory experiences^37,38^, a phenomenon consistent with disinhibition of fronto-temporal connections^39,40^. Disinhibition of language processing centres may result in an inability to distinguish between inner and external speech, resulting in a misattribution of inner speech as externally generated^41–43^. Reduced MWF of temporal white matter tracts observed in this study may reflect a structural deficit underlying this phenomenon. Specifically, reduced MWF is reflective of myelin deficits in these tracts, which is likely to impact speed, reliability and efficiency of nerve signals, thus mediating the failure of this inhibition mechanism. Future studies could usefully explore the association between measures of neural inhibition and myelin content in this specific region. Similarly, abnormal myelination could cause a delay in conducting efference copies of willed inner speech, resulting in corollary discharges being generated too late to suppress the consequences of the inner speech. While we did not observe a correlation between MWF and positive symptom severity in our study, this could be due to the effects of antipsychotic medication on positive symptoms in treatment responsive patients, thus obscuring a true association between symptoms and white matter structure.

Intriguingly, Frith and colleagues observed disinhibition of temporal cortex activation in chronic patients with schizophrenia performing a phonological verbal fluency task^44^. In this study, patients displayed normal frontal activation but an absence of the expected superior temporal gyrus deactivation during word generation, suggesting that dysfunctional connectivity between frontal and temporal regions is a characteristic deficit in schizophrenia. This notion is supported by the finding that healthy controls show focal functional connectivity of the frontal cortex with left-sided temporal language processing areas during a verbal fluency task, whereas never-treated first episode psychosis patients show a more diffuse pattern of frontal connectivity not including these temporal regions^45^. Our findings demonstrate that the structural integrity of temporal tracts may be the key pathology underlying these functional effects, with myelin content the left temporal deep white matter fully mediating verbal fluency differences between patients and controls.

The effects of MWF on verbal fluency are localised within overlapping regions on the left inferior fronto-occipital fasciculus (IFOF) and inferior longitudinal fasciculus (ILF). These fasciculi have been suggested to constitute core aspects, respectively, of the dorsal language processing stream^46^, which is responsible for phonological aspects of language, and the ventral stream^47^, which processes semantic aspects of language. While there is significant overlap between the clusters of association with phonological and semantic verbal fluency in this study, the cluster for phonological verbal fluency is somewhat larger and appears to cover more distinct parts of the IFOF, whereas the semantic verbal fluency cluster could be attributed more to the ILF, in line with their respective roles in language processing. However single-subject tractography would be necessary to ascertain with certainty the exact localisation of these effects.

Verbal fluency has long been a subject of interest in schizophrenia research^48^, with suggestions that it constitutes a primary feature of the illness rather than an epiphenomenon of psychotic symptoms^49^, and is predictive of transition to psychosis in ultra-high risk populations^50^. Deficits in verbal fluency have also been consistently linked with increased levels of negative symptoms^51,52^. Verbal fluency likely reflects both verbal and executive abilities^53^ and as such contributes to more general cognitive functioning. Therefore it is plausible to interpret our findings of an association between left temporal MWF and IQ, but a lack of complete mediation of IQ differences by MWF, in terms of the effects of verbal fluency contributions to overall IQ. Similarly the inverse relationship between left temporal MWF and negative symptoms is in line with the commonly observed association between reduced verbal fluency and negative symptom severity. Negative symptoms have traditionally been associated with dysfunction of the frontal cortex^54^ and diffusion imaging studies have demonstrated that negative symptoms increase with decreased integrity of white matter tracts connecting the frontal cortex to other parts of the brain^55,56^. Recent research has highlighted the importance of accounting for the confounding effects of age on the association between white matter and negative symptoms^57^. All of our analyses controlled for effects of age and gender and the comparison groups did not differ on these variables, rendering a confounding effect unlikely.

We did not observe any associations between myelin content and working memory performance within the temporal region of reduced MWF in psychosis patients. Verbal working memory performance in healthy adults has been associated with fronto-parietal white matter tract integrity in particular^58,59^, reflecting the connecting fibres between grey matter regions most commonly implicated in working memory. In schizophrenia, impaired working memory was shown to be correlated with reduced FA in fronto-parietal connections including the superior longitudinal fasciculus (SLF)^60^, a finding not mirrored in our results. However, we only assessed associations with cognitive measures within the ROI showing reduced MWF in patients compared to controls, a region showing some but not much overlap with the SLF. This was due to our primary aim of assessing specifically the impact of reduced myelin content on cognitive function in psychosis. It is therefore possible that an association exists between working memory and white matter integrity outside of the most affected regions of abnormal myelination in psychosis. Our results suggest that the left temporal white matter region showing abnormal myelin content in patients is not responsible for the working memory deficits observed in these patients. It is possible that working memory deficits are underpinned more strongly by functional neural deficits such as hypoactivation of dorsolateral prefrontal cortex, as demonstrated in seminal works by Weinberger and colleagues^61^.

Previous studies in white matter integrity in patients with a diagnosis of schizophrenia have commonly not only detected disruptions to structural connectivity in left temporal areas, but frequently in more widespread areas throughout the white matter network. Our findings of more focal MWF reductions in left temporal lobe in first episode psychosis patients highlight that abnormalities in this area may occur early on in the illness and represent a causal factor of symptoms rather than downstream effects of chronic illness or medication. In addition, these findings are likely more specific to myelin abnormalities than more widespread findings based on diffusion imaging, which may reflect alterations to other aspects of white matter integrity, such as axonal membrane integrity or fibre density.

This study demonstrates the utility of mcDESPOT for identifying myelin deficits in recent-onset psychosis. We observed significant reductions in myelin content in the left deep temporal white matter, and these reductions fully mediated performance on a verbal fluency task. The lack of association with working memory performance suggests that distinct neural pathologies underlie the deficits seen in these separable cognitive domains. The presence of correlations between abnormal temporal myelination and negative symptoms, which are typically more resistant to conventional antipsychotic medication than are positive symptoms, highlights the importance of identifying therapeutic strategies which could potentially alleviate negative symptomatology by targeting the promotion of myelination early on in the illness.

## Data Availability

The datasets generated during and/or analysed during the current study are available from the corresponding author on reasonable request.
